# Systematics of Scelioninae (Hymenoptera, Platygastroidea): new synonymy, distribution, and species

**DOI:** 10.3897/zookeys.879.38788

**Published:** 2019-10-09

**Authors:** Norman F. Johnson, Luciana Musetti, Lubomír Masner

**Affiliations:** 1 Department of Evolution, Ecology & Organismal Biology, The Ohio State University, 1315 Columbus, Ohio 43212, USA; 2 Department of Entomology, The Ohio State University, Columbus, Ohio 43212, USA; 3 Agriculture and Agri-Food Canada, K.W. Neatby Building, Ottawa, Ontario, Canada

**Keywords:** *
Caloteleia
*, species descriptions, taxonomy, *
Xentor
*

## Abstract

The genera *Doddiella* Kieffer, 1913 and *Aratala* Dodd, 1927 are treated as junior synonyms of of *Aneuroscelio* Kieffer, 1913 following study of the rediscovered holotype of the type species *Aneuroscelio rufipes* Kieffer, 1913 (syn. nov.). The nine species previously recognized in *Doddiella* are all transferred to *Aneuroscelio* (comb. nov.). *Calliscelio schlingeri* (Masner & Johnson) is recognized as a junior synonym of *Calliscelio vitilevuensis* (Fullaway) (**syn. nov.**). *Huddlestonium exu* Polaszek & Johnson is recorded from Kenya, significantly expanding its known range from West Africa (Côte d’Ivoire, São Tomé). A new species of the genus *Tyrannoscelio* Masner, Johnson & Arias-Penna, *T. cerradensis***sp. nov**, is described from Paraguay and the Center-West of Brazil (Mato Grosso). The depositories of the holotypes of five recently described are corrected.

## Introduction

Our knowledge of the diversity of parasitoid wasps in the superfamily Platygastroidea has grown by leaps and bounds over the past 25 years. Since the publication of the last hard-copy taxonomic catalogs for the group (Johnson 1992, Vlug 1995), the number of described genera has grown by 18.5%, from 426 to 505, and the number of species-group taxa has increased an astonishing 68.4%, from 4184 to 7045. The most current online tabulation of the diversity reports 263 valid genera and slightly over 6000 valid species.

In the course of this rapid expansion, several small discoveries and mistakes have been made, most of which would be too minor to merit separate publication. The goal of this contribution is to address these issues and formally document them in the literature.

## Taxonomy

### Status of *Doddiella* Kieffer

In 1913 J.-J. Kieffer published the description of a new genus of scelionine from Aburi in the Gold Coast (present-day Ghana), dedicating it to the teenaged Alan P. Dodd of Queensland. The primary distinguishing characteristic for the new genus was cited as the absence of veins in the wings. This feature was thought to be shared, within the Scelionidae of the time, only with *Rielia* Kieffer, a genus today known as *Mantibaria* Kirby. Ironically, although he was the person intended to be honored, Kieffer's description was not sufficient for Dodd to recognize the genus, and he later described it anew under the name *Aratala* (Dodd 1927). Nine species are currently treated as valid taxa, and the genus is known from the Afrotropical, Oriental, Australian, and Neotropical regions, and also edging into the Palearctic in Egypt and Ethiopia. It is a striking and unmistakable creature, so much so that Masner erected for it the monobasic tribe Doddiellini in 1976. It has received a limited amount of taxonomic attention, having been mentioned in the literature 16 times. Identification keys have been published for the African and Palearctic species (Priesner 1951, Kononova and Kozlov 2008).

In the same year in which *Doddiella* first appeared, Kieffer also described the new genus *Aneuroscelio* from Murang'a (reported as Méranga or Fort-Hall) in British East Africa, modern Kenya. This name languished in obscurity due to the inadequacy of Kieffer's description and lack of study of the single known specimen of the type species, *Aneuroscelio rufipes* Kieffer. The type specimen had not been examined because it was not found in the pinned and mounted collection in the Muséum national d'Histoire naturelle in Paris (see comments in Masner 1976: 56). Through the efforts and kindness of Dr. Claire Villemant of that institution, Kieffer's types from that paper have now been unearthed, preserved in vials of ethanol and kept separate from the rest of the collection. We have since mounted these specimens so that they can be studied and the taxonomic concepts of names they represent can be determined.

**Figures 1–6.**
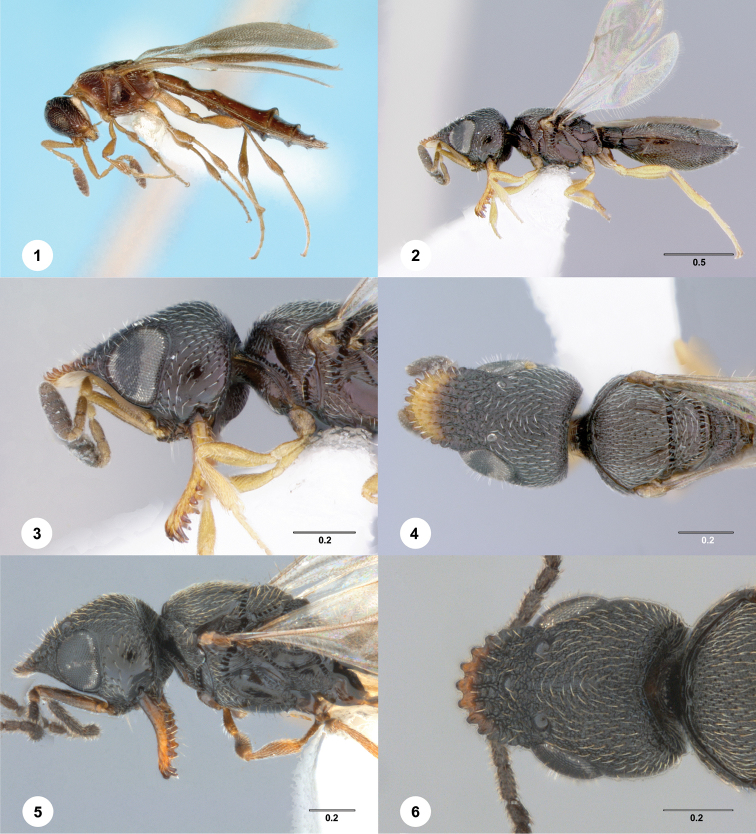
**1** Holotype of *Aneuroscelio rufipes* Kieffer. The flaring of the posterior margins of the metasomal segments is an artifact. **2–4***Tyrannoscelio cerradensis* n.sp., holotype female (OSUC 232307). **2** lateral habitus **3** head and anterior mesosoma, lateral view **4** head and mesosoma, dorsal view. **5–6***T. cerradensis* n.sp., male (OSUC 711174) **5** head and mesosoma, lateral view **6** head, dorsal view. Scale bars in millimeters.

*Aneuroscelio rufipes* is a typical species of *Doddiella* (Fig. 1). Not only does it lack wing veins, but it possesses all of the characteristic features: a dense field of white setae on the gena, mesopleuron smooth and lacking almost all of the the typical sulci and foveae, netrion absent, metascutellum produced into a “blade-like projection," first metasomatic segment elongate, and the posterior margin of the second segment strongly raised and curved (Masner 1976). The two generic names are clearly synonymous. Masner (1976) anticipated this but could not resolve the issue without the type. Beyond the synonymy, the question then is which name is senior?

*Doddiella* was described in the pages of the Bollettino del Laboratorio di Zoologia Generale e Agraria della R. Scuole Superior d'Agricoltura in Portici in volume 7. The index for that volume cites the dates of publication of each article, and Kieffer's paper is dated 20 October 1913. The description of *Aneuroscelio* appeared as a contribution to the Hymenoptera section in "Voyage de Ch. Alluaud et R. Jeannel en Afrique Orientale (1911-1912)", and an insert in that book dates the article to 15 August 1913. Thus, the name *Aneuroscelio* has priority over the much better known *Doddiella*.

#### Aneuroscelio

Taxon classificationAnimaliaHymenopteraPlatygastroidea

Kieffer

31007752-E748-5982-8B74-1A68E8DAC9B6

Aneuroscelio Kieffer, 1913a: 14. Type: Aneuroscelio rufipes Kieffer, by monotypy and original designation. Kieffer, 1926: 266, 278 (description, keyed); Muesebeck & Walkley, 1956: 328 (citation of type species); Johnson, 1992: 336 (cataloged, catalog of world species).
Doddiella Kieffer, 1913b: 109. Type: Doddiella nigriceps Kieffer, by monotypy and original designation. **Syn. nov**. Kieffer, 1926: 266, 281 (description, keyed); Priesner, 1951 (key to African species); Muesebeck & Walkley, 1956: 348 (citation of type species); Masner, 1976: 6, 56 (description, keyed, synonymy); Galloway & Austin, 1984: 5, 77 (diagnosis, list of species described from Australia, keyed); Johnson, 1992: 367 (cataloged, catalog of species); Austin & Field, 1997: 36, 68 (structure of ovipositor system, discussion of phylogenetic relationships); Lê, 2000: 31, 87 (keyed, description); Rajmohana K., 2006: 115 (keyed); Kononova & Kozlov, 2008: 21, 181 (description, keyed, key to species of Palearctic region); Rajmohana, 2014: 6, 21 (description, keyed).
Aratala Dodd, 1927: 74. Type: Aratala globiceps Dodd, by monotypy and original designation. **Syn. nov**. Muesebeck & Walkley, 1956: 331 (citation of type species); Masner, 1976: 56 (junior synonym of Doddiella Kieffer).

### List of species

*Aneuroscelio aegyptiacus* (Risbec, 1950), **comb. nov.**

= Aneuropria aegyptiaca var. microcephala Risbec, 1954

*Aneuroscelio dolabella* (Kozlov & Lê, 1986), **comb. nov.**

*Aneuroscelio globiceps* (Dodd, 1927), **comb. nov.**

*Aneuroscelio indicus* (Mukerjee, 1993), **comb. nov.**

*Aneuroscelio kiefferi* (Priesner, 1951), **comb. nov.**

*Aneuroscelio maindroni* (Risbec, 1955), **comb. nov.**

*Aneuroscelio nigricephala* (Mukerjee, 1993), **comb. nov.**

*Aneuroscelio nigriceps* (Kieffer, 1913b), **comb. nov.**

*Aneuroscelio rufipes* Kieffer, 1913a

*Aneuroscelio similis* (Priesner, 1951), **comb. nov.**

### Status of *Xentor schlingeri* Masner & Johnson

We described the genus *Xentor* in 2007 for three quite distinctive species from Fiji: *X. schlingeri* Masner & Johnson, *X. filicornis* Masner & Johnson, and *X. convexifrons* Masner & Johnson. On the basis of newly discovered characters, Talamas et al. (2016) synonymized *Xentor* under *Calliscelio* Ashmead, a speciose and cosmopolitan genus. We have later independently corroborated this hypothesis with molecular evidence (*unpublished data*). Accordingly, the three species of *Xentor* were transferred to *Calliscelio*. During a visit to the J. Linsley Gressitt Center for Research in Entomology at the Bernice Pauahi Bishop Museum (Honolulu) we discovered that the most distinctive species and the type species of *Xentor*, *C. schlingeri*, had already been described by D.T. Fullaway under the name *Caloteleia vitilevuensis*Fullaway (1939). Thus, the name *Xentor schlingeri* falls as a junior synonym of *Calliscelio vitilevuensis* (Fullaway), syn. nov.

### Corrections of holotype depositories

The collections in which the holotypes for the following species are deposited were reported incorrectly. The corrections are noted alongside the taxon name.

*Axea atai* Valerio & Yoder: The Natural History Museum, London, UK

*Axea dorothae* Valerio & Yoder: The Natural History Museum, London, UK

*Axea mwari* Valerio & Yoder: Nairobi National Museum, Nairobi, Kenya

*Oreiscelio magnipennis* Talamas: Nairobi National Museum, Nairobi, Kenya

*Paridris trispinosa* Talamas & Masner: The Natural History Museum, London, UK

### *Huddlestonium exu* Polaszek & Johnson is widespread in Africa

The genus *Huddlestonium* is a curious creature whose features demand an expansion of the boundaries of what is morphologically possible in the Platygastroidea (Masner et al. 2007). It clearly belongs to the superfamily as it possesses the characteristic ventral papillar sensilla on the apical claval segments of the female (the male is, as yet, undiscovered). However, it has no well-developed laterotergites and laterosternites on the metasoma and the female antenna is uniquely 13-merous. It was described from two collections, a single specimen from the Côte d'Ivoire and a short series of four specimens from the island of São Tomé, both collecting localities in western Africa. Among the extant fauna, it is most similar to the Neotropical genus *Plaumannion*, a group that is even rarer than *Huddlestonium* as it is known from only 3 specimens (one of which is broken). In terms of the fossil record, *Huddlestonium* bears a striking resemblance to the Eocene genus *Archaeoscelio* Brues (see Masner et al. 2007) and perhaps even to the recently described Cretaceous species *Geoscelio mckellari* Engel & Huang (Engel et al. 2017).

It was, therefore, of some surprise to find new specimens of *Huddlestonium* collected nearly 2000 miles east of São Tomé in western Kenya. One specimen (UCRC ENT 154639) was collected in Isecheno Nature Reserve (0.24°N, 34.87°E); and two (OSUC 192430, 232305) in Ruma National Park (0.65°S, 34.33°E). The specimens differ slightly from their west African counterparts, particularly in the closer proximity of the lateral ocelli to the margins of the compound eyes. We were initially tempted to treat these specimens as a new species. Despite the great distance separating the collecting localities, the morphological differences seem too slight to warrant that course of action, particularly given the small number of specimens at hand. The new data do indicate that *Huddlestonium* is much more widely distributed than previously known. Unfortunately, we remain ignorant of the hosts that they parasitize.

### A new species of *Tyrannoscelio* Masner, Johnson & Arias-Penna

The genus *Tyrannoscelio* is known from only two species: *T. genieri* Masner & Johnson from the southeastern Brazilian state of Espírito Santo, and *T. crenatus* Arias-Penna, known from two specimens from the opposite side of the continent, in the Colombian province of Caquetá. The genus is immediately recognizable on the basis of the expanded, crenellated frontal shelf, and the extraordinarily elongate mandibles. More subtly, though, the genus is notable for the presence of a distinct skaphion and the lack of a postmarginal vein in the forewing. Here we describe a third species of the genus, from central Paraguay and the Brazilian state of Mato Grosso.

#### Tyrannoscelio cerradensis sp. nov.

Taxon classificationAnimaliaHymenopteraPlatygastroidea

8E0EA38D-C638-5B53-BF8B-416C9490EACD

http://zoobank.org/D7781AC6-EB44-4863-BFAC-8FA15FE24A21

Figs 2–6

##### Diagnosis.

Similar to other known species in the genus, differing in the following characters. Body color: entirely dark brown except for brownish-yellow apex of frontal shelf. Frontal shelf: margined by 13–14 rounded teeth in female, ten in male. Median longitudinal furrow on vertex: weak, incomplete, visible only near occipital carina. Sculpture of vertex: rugose-reticulate, with superimposed coriaceous microsculpture. OOL: slightly less than ocellar diameter. Outer margin of mandible: with five to six teeth. Sculpture of mesoscutum: coriaceous, with longitudinal striae present only near transscutal articulation. Sculpture of mesoscutellum: rugose, with superimposed coriaceous microsculpture. Notauli: present only in posterior half of mesoscutum. Metascutellum shape: roughly triangular. Mesopleural carina: distinct, complete. Plicae on propodeum: well-developed. Felt field: present on S2.

##### Material еxamined.

Holotype female: OSUC 232307, PARAGUAY: San Pedro, Cororo-Rio Ypane, XII-5/9-1983, Malaise Trap, M. Wasbauer coll. Deposited in California Department of Food and Agriculture (Sacramento). Paratypes: 3 males, OSUC 711174, 786576, 786579. BRAZIL: Mato Grosso, Fazenda Formozinho, Mun. Tangará da Serra, 594 m, 14°29'33"S 57°55'49"W, 14.xii.2013, cerradão, flight int. trap, F. Génier & L. Sawaris, 2013-152 (CNCI).

##### Etymology.

The specific epithet refers to the cerrado habitat in which the specimens were collected and is treated as an adjective.

##### Comments.

Since the original description of *T. genieri* several additional specimens have been collected in the Brazilian state of Espírito Santo in or near the Sooretama Biological Reserve, the same area from which the species was described originally. The habitats are described on the specimen labels as semi-deciduous or primary lowland Atlantic forest. The additional species in the Center-West of Brazil, Paraguay, and Colombia suggests that the genus is very widely distributed and rare or perhaps restricted in its habitat preferences or timing of adult emergence.

### Key to species of *Tyrannoscelio*

**Table:** 

1	Outer edge of mandible with three teeth near apex; mesosoma lighter in color than head and mesosoma; mesoscutum longitudinally rugose throughout (southeast Brazil)	***T. genieri***
–	Outer edge of mandible with five to six teeth along its entire length; from dorsal view head, mesosoma, metasoma all dark brown; longitudinal sculpture on mesoscutum limited at most to area near transscutal articulation	**2**
2	Metascutellum tridentate, lateral teeth distinct; mesoscutellum coriaceous (Colombia)	***T. crenatus***
–	Metascutellum triangular, without lateral teet; mesoscutellum with irregular longitudinal rugulae with superimposed coriaceous microsculpture (central-west Brazil, Paraguay)	***T. cerradensis***

## Supplementary Material

XML Treatment for Aneuroscelio

XML Treatment for Tyrannoscelio cerradensis
